# Half-dose verteporfin photodynamic therapy for bullous variant of central serous chorioretinopathy: a case report

**DOI:** 10.1186/1752-1947-5-208

**Published:** 2011-05-26

**Authors:** Winnie WK Ng, Zenith HY Wu, Timothy YY Lai

**Affiliations:** 1 Department of Ophthalmology & Visual Sciences, Hong Kong Eye Hospital, The Chinese University of Hong Kong, Hong Kong

## Abstract

**Introduction:**

Central serous chorioretinopathy is characterized by serous neurosensory detachment of the macula and it usually resolves spontaneously with good visual prognosis. In some patients, however, the serous retinal detachment might be very extensive and can result in bullous exudative retinal detachment. We evaluated the use of half-dose verteporfin photodynamic therapy for the treatment of bullous retinal detachment in idiopathic central serous chorioretinopathy.

**Case presentation:**

We report the case of a 51-year-old Chinese man who presented with blurred vision in his right eye and superior visual field defect due to bullous variant of central serous chorioretinopathy. No improvement in vision and retinal detachment was noted after three months of observation and a short course of oral acetazolamide. He was then treated with half-dose verteporfin photodynamic therapy and his visual acuity improved from 20/70 to 20/25 within one month of treatment. Three months after photodynamic therapy, there was complete resolution of sub-retinal fluid and bullous retinal detachment. No recurrence of central serous chorioretinopathy was noted in three years of follow-up.

**Conclusion:**

We report the beneficial effect of photodynamic therapy with half-dose verteporfin as a treatment option for bullous retinal detachment caused by central serous chorioretinopathy.

## Introduction

Central serous chorioretinopathy (CSC) is characterized by serous neurosensory detachment of the macula and it usually resolves spontaneously with good visual prognosis. In some patients, however, the serous retinal detachment might be very extensive and a large amount of sub-retinal fluid can result in bullous exudative retinal detachment [1]. Photodynamic therapy (PDT) with verteporfin has been shown to be effective in the treatment of CSC but the use of conventional dosage of verteporfin (6 mg/m^2^) might be associated with complications such as iatrogenic choroidal neovascularization, diffuse retinal epithelial atrophy and more severe retinal thinning [2-4]. We report the use of half-dose (3 mg/m^2^) verteporfin PDT for treating a patient with the severe bullous form of CSC.

## Case Presentation

**Figure 1:**
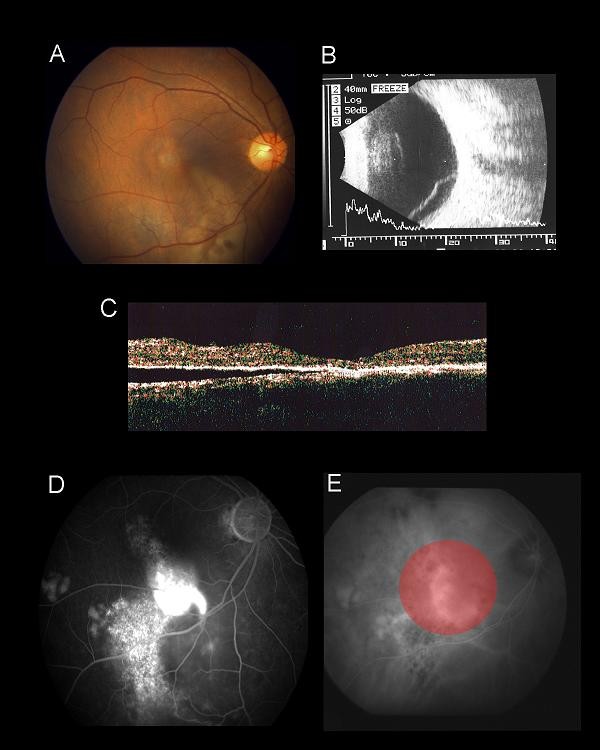
**(A) Fundus photo of his right eye at presentation showing exudative retinal detachment at the macula and inferior retina**. Fibrinous exudate can be seen at the inferior arcade. **(B) **B-scan ultrasound axial scan showing inferior exudative retinal detachment. **(C) **OCT imaging of the right macula showing the presence of sub-retinal fluid involving the fovea. **(D) **Mid-phase FA showing diffuse fluorescein leakage at the macula with RPE track inferiorly. **(E) **Mid-phase ICGA showing dilated choroidal vasculature with hyperdynamic circulation consistent with central serous chorioretinopathy. Laser for PDT was applied to the area of choroidal hyperpermeability as guided by ICGA (red circle).

A 51-year-old Chinese man with good past health presented with a 10 day history of reduced vision and a superior visual field defect of the right eye. He denied a history of recent steroid use via any route. His best-corrected visual acuity was 0.5 OD and 1.0 OS. An examination of the fundus showed inferior bullous retinal detachment in his right eye with yellowish fibrinous exudates and retinal pigment epithelial (RPE) changes without retinal break (Figure 1A). An examination of the anterior segment and vitreous cavity showed an absence of any inflammatory reaction suggestive of an inflammatory cause such as Vogt-Koyanagi-Harada disease. A B-scan ultrasound confirmed inferior retinal detachment (Figure 1B) and optical coherence tomography (OCT) showed foveal involvement (Figure 1C). Fluorescein angiography (FA) revealed early hyper-fluorescence with diffuse late leakage at the macula (Figure 1D) and indocyanine green angiography (ICGA) showed dilated choroidal vessels with choroidal hyper-perfusion consistent with CSC (Figure 1E). An examination of the fundus of his left eye showed mild RPE changes at the superior macula, with a mild RPE window defect on FA and mildly dilated choroidal vessel on ICGA. The findings in his left eye were consistent with resolved CSC.

**Figure 2:**
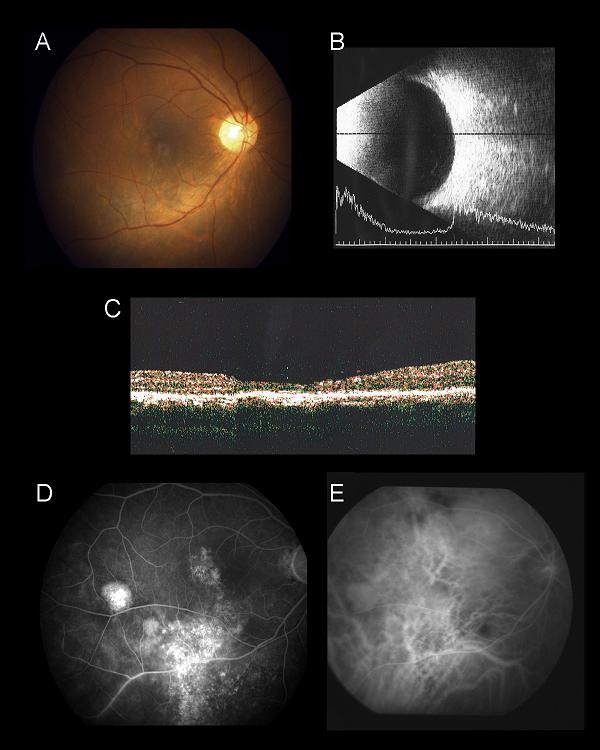
**(A) Fundus photo of his right eye three months after half-dose verteporfin PDT showing complete resolution of the macular and inferior retinal detachments**. **(B) **B-scan ultrasound axial scan showing resolution of the inferior exudative retinal detachment. **(C) **OCT imaging of the right macula showing absence of sub-retinal fluid at the macula with thinning of the neurosensory retina due to CSC. **(D) **Mid-phase FA showed diffuse RPE window defect with staining due to sub-retinal fibrosis. **(E) **Mid-phase ICGA showing absence of dilated choroidal vasculature.

A diagnosis of bullous CSC was made and despite a two-week course of oral acetazolamide (250 mg qid) and observation for three months, the exudative retinal detachment persisted and his right eye vision deteriorated to 0.3. He subsequently underwent half-dose (3 mg/m^2^) verteporfin PDT with a spot size of 4500 μm to cover the area of dilated choroidal vessels in ICGA. One month after the PDT, his vision improved to 0.8 OD with a reduction in inferior retinal detachment. After three months, there was complete absence of sub-retinal fluid (Figure 2A). A B-scan ultrasound and OCT confirmed the resolution of exudative retinal detachment (Figures 2B and 2C). FA and ICGA showed reduced leakage and choroidal hyperpermeability (Figures 2D and 2E). He was followed for 38 months, during which there was no recurrence and his final vision was 1.0 OD.

## Discussion

Bullous CSC is an uncommon form of CSC associated with a large amount of sub-retinal fluid. The visual prognosis of bullous CSC is generally poorer than those limited to the macula, as recurrence is common even after initial complete regression [1]. Traditional management options of bullous CSC include observation or thermal laser photocoagulation. However, previous studies have shown that the outcome of thermal laser photocoagulation is similar with the natural course of the disease in terms of disease duration and final visual acuity [1].

In recent years, PDT with verteporfin has been utilized for treating patients with CSC. However, full-dose PDT for treating CSC is not without complications, as retinal pigment epithelium atrophy, retinal thinning and choroidal neovascularization have been reported after PDT for chronic CSC [2-4]. In order to decrease the extent of collateral damage and the risk of adverse events, a reduced dose of verteporfin has been used to treat CSC, with the efficacy of PDT remained high [3,5,6].

To the best of our knowledge, the long-term outcome of half-dose PDT for bullous variant of CSC has not previously been reported. Our case demonstrated that this treatment is effective in reducing the bullous exudative retinal detachment and improving the patient's vision rapidly. Moreover, there was no evidence of any disease recurrence or complications after more than three years of follow-up. The reasons for the successful treatment outcome might be related to the idiopathic cause of the disease and intuition of early treatment in our patient. Although the longer term complications and recurrence rate remains unknown, half-dose verteporfin PDT for bullous CSC appeared to be an effective treatment option and might be considered as a management option for these patients.

## Conclusion

Bullous exudative retinal detachment is an uncommon manifestation of CSC and can result in significant visual loss. Our patient demonstrated that half-dose verteporfin PDT is an effective treatment option for bullous CSC, resulting in rapid resolution of exudative retinal detachment and improvement in vision.

## Consent

Written informed consent was obtained from the patient for publication of this manuscript and any accompanying images. A copy of the written consent is available for review by the Editor-in-Chief of this journal.

## Competing interests

The authors declare that TYYL has received honorium for lecture fees and serving in the advisory board of Novartis Pharmapeutical Inc. The other authors (WWKN, ZHYW) have no competing interests.

## Authors' contributions

WWKN interpreted the patient data and wrote the first draft of the manuscript. ZHYW had a role in writing a final draft of the manuscript and in the preparation of clinical images. TYYL performed the treatment and follow-up on the patient, and was a major contributor to the writing of the manuscript. All authors read and approved the final manuscript.
